# Astaxanthin improves the development of the follicles and oocytes through alleviating oxidative stress induced by BPA in cultured follicles

**DOI:** 10.1038/s41598-022-11566-1

**Published:** 2022-05-12

**Authors:** Yaqiu Li, Zhu Dong, Sitong Liu, Fan Gao, Jinyu Zhang, Zhendong Peng, Lixin Wang, Xiaoyan Pan

**Affiliations:** 1 Center for Reproductive Medicine, Jilin Medical University, No. 5, Jilin Street, Jilin, 132013 China; 2 Department of Anatomy, Jilin Medical University, Jilin, 132013 China

**Keywords:** Physiology, Medical research

## Abstract

This study is to investigate whether astaxanthin could alleviate the oxidative stress damages of follicles induced by BPA and improve the development of the cultured follicles and oocytes. Compared with BPA group, the survival rate, antrum formation rate, oocyte maturation rate and adherence area of the D8 and D10 follicles of the BPA+Asta group were significantly higher. The estrogen and progesterone in the culture medium of BPA+Asta group were significantly higher. PCNA in D8 and D10 granulosa cells and ERα in D10 granulosa cells of follicles in BPA+Asta group were significantly higher. The levels of malondialdehyde in the follicle culture medium, levels of ROS in the oocytes, the expression levels of *caspase 3* and *cathepsin B* in the oocytes of the BPA+Asta group were significantly lower. However, the mitochondrial membrane potential, and the expression levels of antioxidant genes (*CAT, SOD1* and *SOD2*) and anti-apoptotic gene *Bcl-2* in the oocytes in the BPA+Asta group were significantly higher. Astaxanthin improves the development of follicles and oocytes through increasing the antioxidant capacity of follicles and oocytes, and relieving the BPA-induced oxidative stress during follicular development and oocyte maturation.

## Introduction

Bisphenol A (BPA) is an industrial raw material that has been widely used for the production of polycarbonate plastics and epoxy resins. BPA represents one of the most produced and widely used chemicals in the world, with global consumption of 7.7 million tons in 2015 and a projected consumption of 10.6 million tons by 2022^1^. People are widely exposed to BPA-containing environments, and BPA has been detected in packaged food, drinking water, air, and dust particles^2,3^. In 86% of the indoor dust samples, BPA levels range from 0.2 to 17.6 μg/g. In urban outdoor environments, BPA has been detected at an average level of 0.51 ng/m^3^ in air samples, while BPA is detected at an average level of 208 ng/m^3^ in air samples from plastic factories^4^. BPA in the environment would enter the human body through breathing, drinking water, diet and skin contact, etc. In the biomonitoring study of human blood/serum samples, unmetabolized BPA has been found to be stable in the concentration range of 0.5–3 ng/Ml^4^. The concentration of BPA in the blood/serum samples of the workers in the plastic product factory is significantly higher^5^. Moreover, the concentration of BPA in the blood/serum samples of the workers who work more than 5 years (27.18 ng/mL) is significantly higher than the workers who work less than 5 years (9.73 ng/mL). Unmetabolized BPA would accumulate in the human body for a long time. As an endocrine and metabolism disruptor, BPA could cause adverse effects on the health of humans and animals^6–9^. It can increase the mediators of oxidation reactions and reduce the production of antioxidant enzymes^10^. Additionally, it significantly increases the lipid peroxidation and reactive oxygen species (ROS) levels in neuroblastoma cells^11^, male germ cells^12^, intestinal epithelial cells^13^, and kidney tubular cells^14^, causing mitochondrial dysfunction and changes in intracellular signaling pathways, inducing cell apoptosis and causing oxidative stress damages to the immune system, nervous system, reproductive system and digestive system^10–14^. One clinical study has found that the high concentration of BPA in the urine of infertile women was clearly related to the decreased number of primordial follicles^15^. More and more laboratory data have also shown that long-term exposure to BPA can damage the germ cells and affect the reproductive function^12,16–18^.

In recent years, many antioxidants have been used to alleviate the oxidation reaction caused by BPA and inhibit the production of excessive ROS, such as vitamin C, vitamin E, N-acetylcysteine, lipoic acid, ginger extract and gallic acid, etc.^19–23^. These antioxidants have certain protective effects on sperm motility and sperm morphology, and can reduce the oxidative stress and apoptosis caused by BPA to varying degrees^24^. The 1,25-dihydroxyvitamin D3 also has a significant alleviating effect on the oxidative stress and mitochondrial damages in ovarian granulosa cells caused by BPA^25^. However, the effects and mechanisms of antioxidants on follicles and oocytes exposed to BPA are still unclear.

Astaxanthin is a carotenoid found in aquatic products (salmon, shrimp, and crab, etc.)^26^. It has stronger antioxidant properties than vitamin C, vitamin E and β-carotene. For example, its antioxidant capacity is 100–500 times that of α-vitamin E and 15 times that of carotenoids^26^. It can reduce lipid peroxidation and eliminate excess ROS^27^. Since astaxanthin is fat-soluble, it could enter the cell membrane and reduce the DNA damage^26^. Astaxanthin can induce the expression of antioxidant genes and inhibit the expression of apoptosis genes during the in vitro bovine embryo development^28^, thereby increasing the development rate of embryos treated with heat stress^29^. Astaxanthin could promote the oocyte maturation and fertilization of the in vitro cultured heat-stressed porcine oocytes^30^. Moreover, astaxanthin could reduce the content of ROS and cathepsin B gene expression in oocytes and promote the oocyte maturation of bovine early antral follicles through its antioxidant effects^31^. Astaxanthin can alleviate the oxidative damages of heat stress to oocytes and embryos^28–30^. It is unknown whether astaxanthin could alleviate the oxidative stress damages of BPA to follicles.

In this study, the in vitro cultured mouse preantral follicles were treated with BPA and astaxanthin, and the effects and mechanisms of astaxanthin on BPA-induced oxidative stress damage to follicles were investigated. Our findings might provide the experimental and theoretical basis for developing the anti-oxidative drugs for the protection of female reproductive system.

## Results

### Astaxanthin alleviates inhibitory effects of BPA on follicular development and oocyte maturation

**Figure 1:**
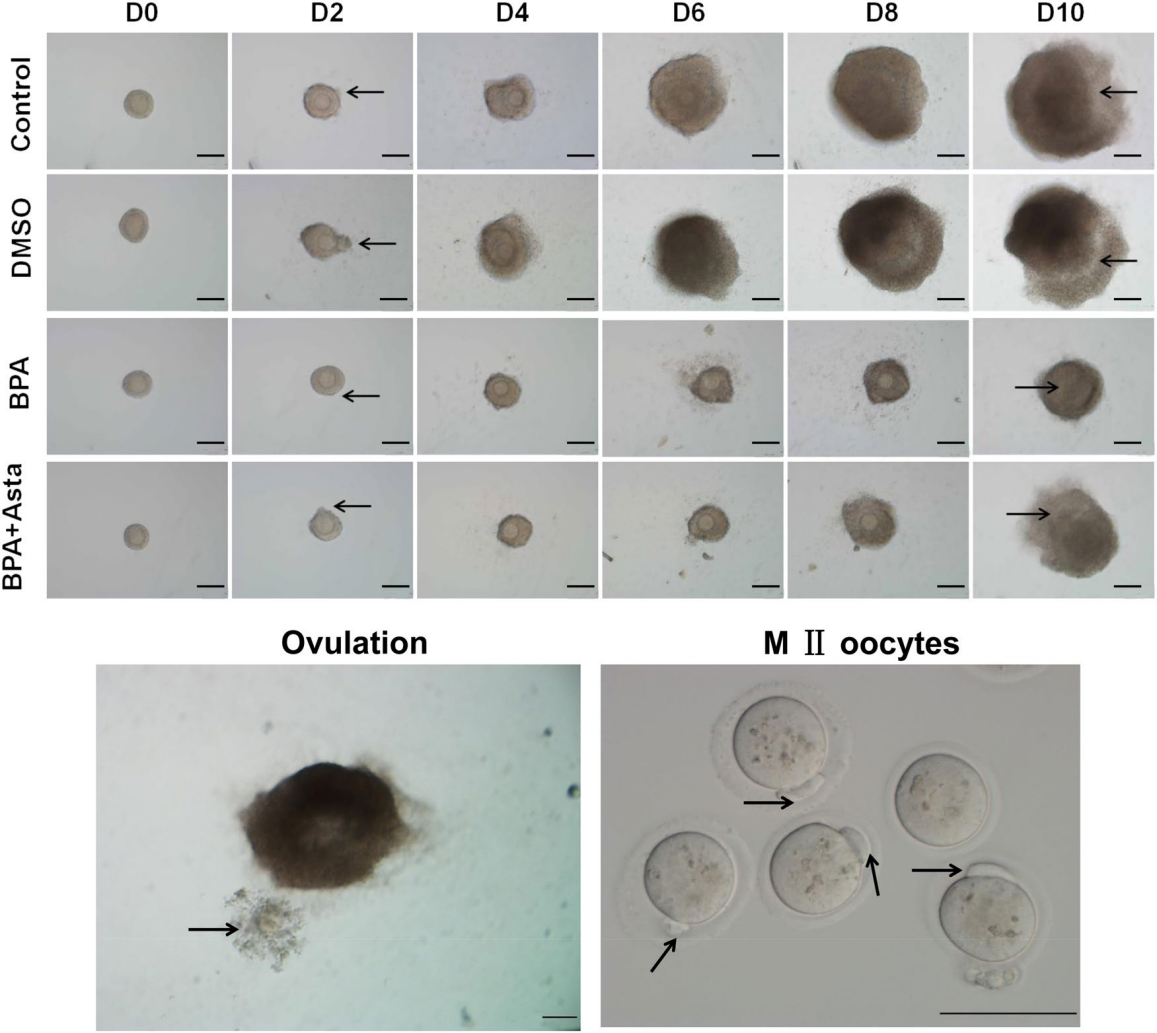
The in vitro development of follicles at various stages (Scale bar, 100 mµ). On D2, the arrow head indicated the outgrowth of the adhered follicular cells; On D10, the arrow head indicated the follicular antrum; In the picture of ovulation, the arrows indicated the ovulated cumulus-oocyte complexes (CoCs); and in the picture of M II oocytes, the arrows indicated the first polar body excreted by mature oocytes.

Preantral follicles were cultured in vitro for 11 d. The developmental status of the follicles were observed on D2, D4, D6, D8 and D10 (Fig. 1). In the control group and DMSO group, on D2, the follicles begin to grow adherently, and the follicular theca cells grew outward. Subsequently, as the granulosa cells proliferated, the volume of the follicle gradually was increased. On D10, the follicle antrum was observed. At 14–16 h after adding hCG, mature follicles ovulated. In the BPA group, on D2, a few follicles did not grow adherently and died on the following days. From D6 to D10, the follicles were smaller than those in the control group and DMSO group. On D10, antrum could not be formed in a few follicles. In the BPA+Asta group, from D2 to D10, the development of the follicles was similar to that in the control group and DMSO group. The follicle development rates of these groups were analyzed and shown in Table 1. Compared with the control group, the survival rate, antrum formation rate and oocyte maturation rate of the BPA group were significantly decreased. The survival rate and antrum formation rate of the follicles, and the maturation rate of oocytes, in the BPA+Asta group, were significantly higher than the BPA group. The 0.2% DMSO showed no significant effects on the development rate of follicles at each stage. There was no significant difference in the ovulation rate of follicles among these groups. These results suggest that, astaxanthin could attenuate the inhibitory effects of BPA on follicular development and oocyte maturation.

**Table 1:** The in vitro development of mouse follicles.

Group	Number of cultivated oocytes	Survival rate (%)	Antrum formation rate (%)	Ovulation rate (%)	Maturation rate (%)
Control	180	179 (99.44 ± 0.96)	154 (86.01 ± 4.36)	146 (94.76 ± 1.36)	120 (82.28 ± 1.92)
DMSO	180	178 (98.89 ± 1.93)	154 (86.44 ± 6.39)	145 (94.24 ± 1.46)	118 (81.51 ± 2.86)
25 µM BPA group	180	151 (83.89 ± 5.95^a^)	108 (71.43 ± 2.29^a^)	100 (92.57 ± 1.69)	46 (45.81 ± 2.68^a^)
25 µM BPA + 2.5 nM Asta group	180	170 (94.44 ± 2.55^b^)	142 (83.45 ± 7.46^b^)	133 (93.46 ± 2.85)	98 (74.09 ± 4.52^ab^)

Compared with control, ^a^*P* < 0.05; and compared with the 25 µM BPA group ^b^*P* < 0.05.

### Astaxanthin promotes granulosa cell proliferation

**Figure 2:**
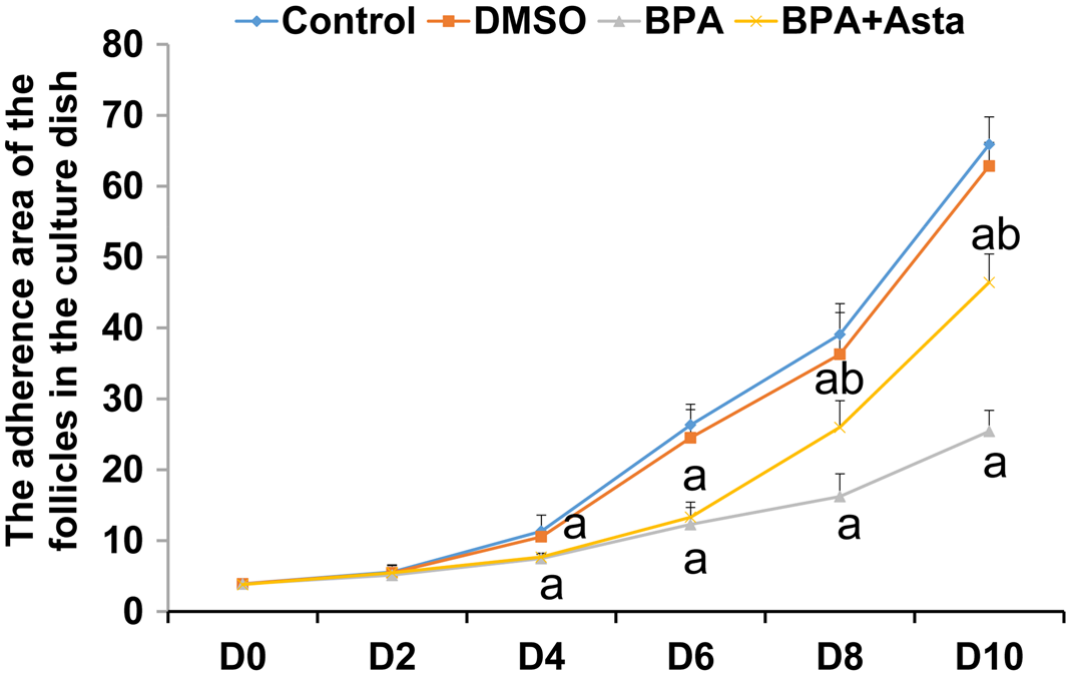
Analysis of adherence area of follicle growth (N = 10). Compared with control, ^a^ *P* < 0.05; and compared with BPA group, ^b^*P* < 0.05.

The adherence area of D2, D4, D6, D8 and D10 follicles were observed and analyzed. As shown in Fig. 2, compared with the control group, the adherence area of the D4, D6, D8 and D10 follicles was significantly reduced in the BPA group (*P* < 0.05). The adherence area of the D2, D4 and D6 follicles in the BPA+Asta group was not significantly different from the BPA group, while that of the D8 and D10 follicles in the BPA+Asta group was significantly larger than the BPA group (*P* < 0.05) (Fig. 2). The enlargement of the adherence area indicated the proliferation of the granulosa cells from the follicle. Astaxanthin increased the adherence area of the follicles on D8 and D10, indicating that it promoted the proliferation of the granulose cells from the follicles on D8 and D10.

**Figure 3:**
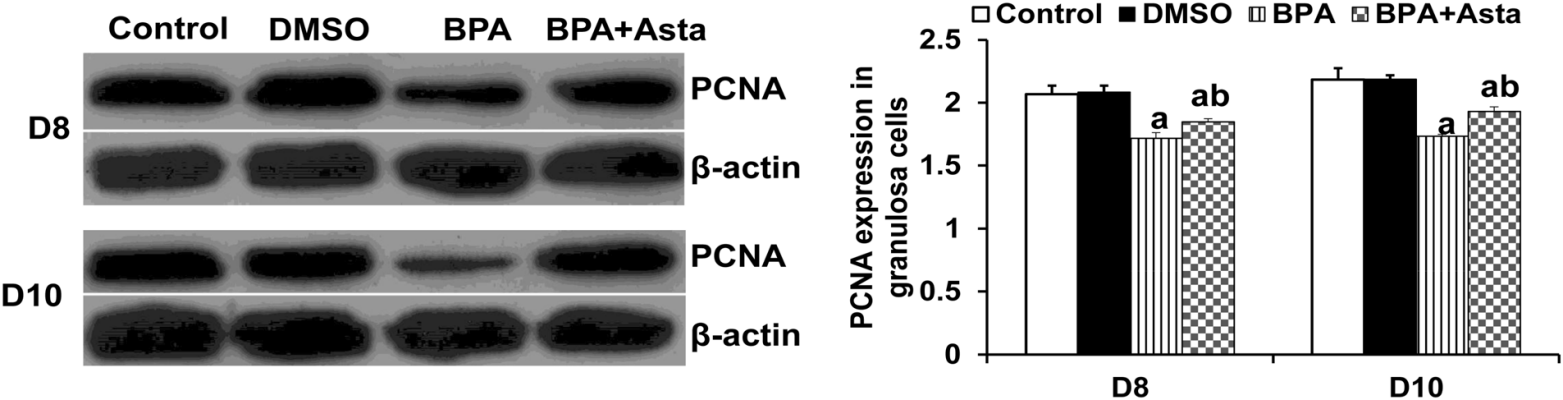
PCNA expression levels in granulosa cells. The expression levels of PCNA in D8 and D10 follicular granulosa cells were detected with Western blot analysis. Compared with control, ^a^ *P* < 0.05; and compared with BPA group, ^b^ *P* < 0.05.

Western blotting was performed to detect the expression of PCNA, which is an indicator of cell proliferation. As shown in Fig. 3, compared with the control group and the DMSO group, the PCNA expression levels in the D8 and D10 granulosa cells in the BPA group were significantly reduced (*P* < 0.05). The PCNA expression levels of D8 and D10 granulosa cells in the BPA+Asta group were significantly higher than the BPA group (*P* < 0.05), which was still significantly lower than the control group (*P* < 0.05) on D8 and D10. These results suggest that astaxanthin could increase the expression of PCNA in granulosa cells, thus promoting their proliferation.

### Astaxanthin improves secretion of estrogen and progesterone and expression of ERα in granulosa cells

**Figure 4:**
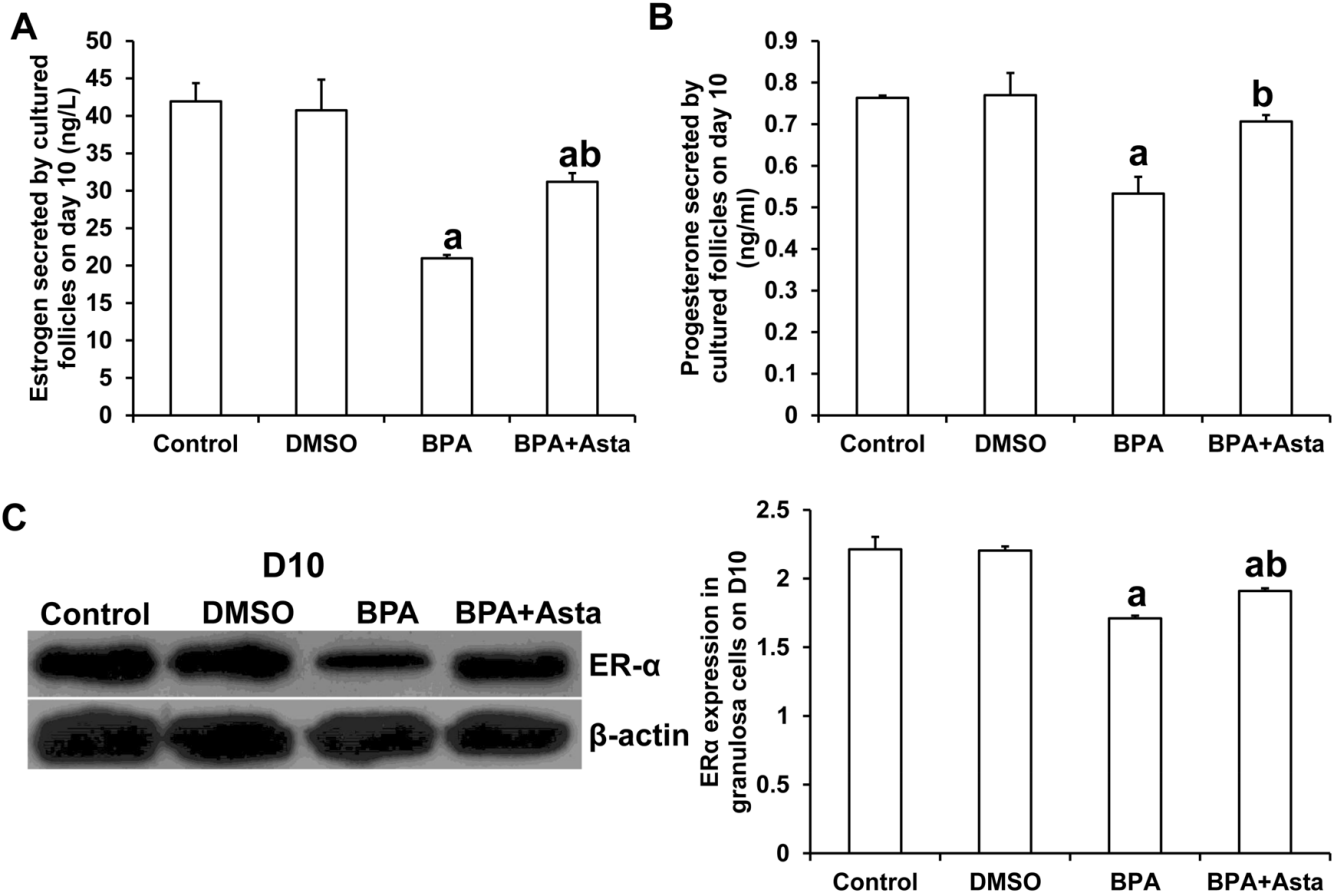
Estrogen and progesterone contents and ERα expression level in cultured D10 follicles. (**A**) The secretion levels of estrogen by D10 follicles (ng/L). (**B**) The secretion levels of progesterone by D10 follicles (ng/ml). (**C**) The expression levels of ERα in D10 follicular granulosa cells were detected with Western blot analysis. Compared with control, ^a^*P* < 0.05; and compared with BPA group, ^b^*P* < 0.05.

The culture medium of D10 follicles was collected separately, and the levels of estrogen and progesterone secreted by the follicles were measured by ELISA. As shown in Fig. 4, compared with the control group, the levels of estrogen and progesterone in the culture medium of the BPA group were significantly reduced (*P* < 0.05) (Fig. 4A and B). The levels of estrogen and progesterone in the culture medium of the BPA+Asta group were higher than those in the BPA group (*P* < 0.05). These results suggest that astaxanthin increases the secretion of estrogen and progesterone in D10 follicular granulosa cells.

The granulosa cells of D10 follicles were collected separately for Western blotting analysis of ERα. As shown in Fig. 4C, compared with the control group, the expression level of ERα in the granulosa cells in the BPA group and BPA+Asta group was significantly reduced (P < 0.05) (Fig. 4C). However, the expression level of ERα in the granulosa cells in the BPA+Asta group was significantly higher than the BPA group (*P* < 0.05). These results suggest that astaxanthin stimulates the expression of ERa in granulosa cells in the late stage of follicular development.

### Astaxanthin reduces malondialdehyde (MDA) and ROS levels in follicle culture medium and oocytes

**Figure 5:**
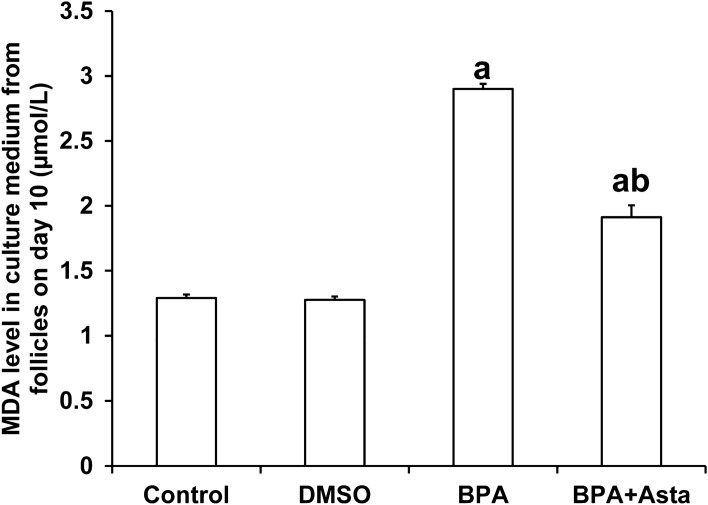
MDA contents in D10 follicle culture in vitro (µmol/L) (N = 10). Compared with control, ^a^*P* < 0.05; and compared with BPA group, ^b^*P* < 0.05.

MDA is a metabolite of membrane lipid peroxidation. The content of MDA in the culture medium reflects the damage degree to the cell membrane of follicle cells after oxidative stress, which is closely related to the developmental potential of the follicle^32^. In this study, the D10 follicle culture medium was collected and subjected to the MDA detection. As shown in Fig. 5, compared with the control group, the levels of MDA in the follicular culture medium of the BPA group and the BPA+Asta group were significantly increased (*P* < 0.05). After astaxanthin treatment, MDA level in the culture medium of the BPA+Asta group was significantly reduced than that of the BPA group (*P* < 0.05). These results suggest that astaxanthin protects cell membranes of the cultured follicles from lipid peroxidation through reducing the production of MDA by follicles.

**Figure 6:**
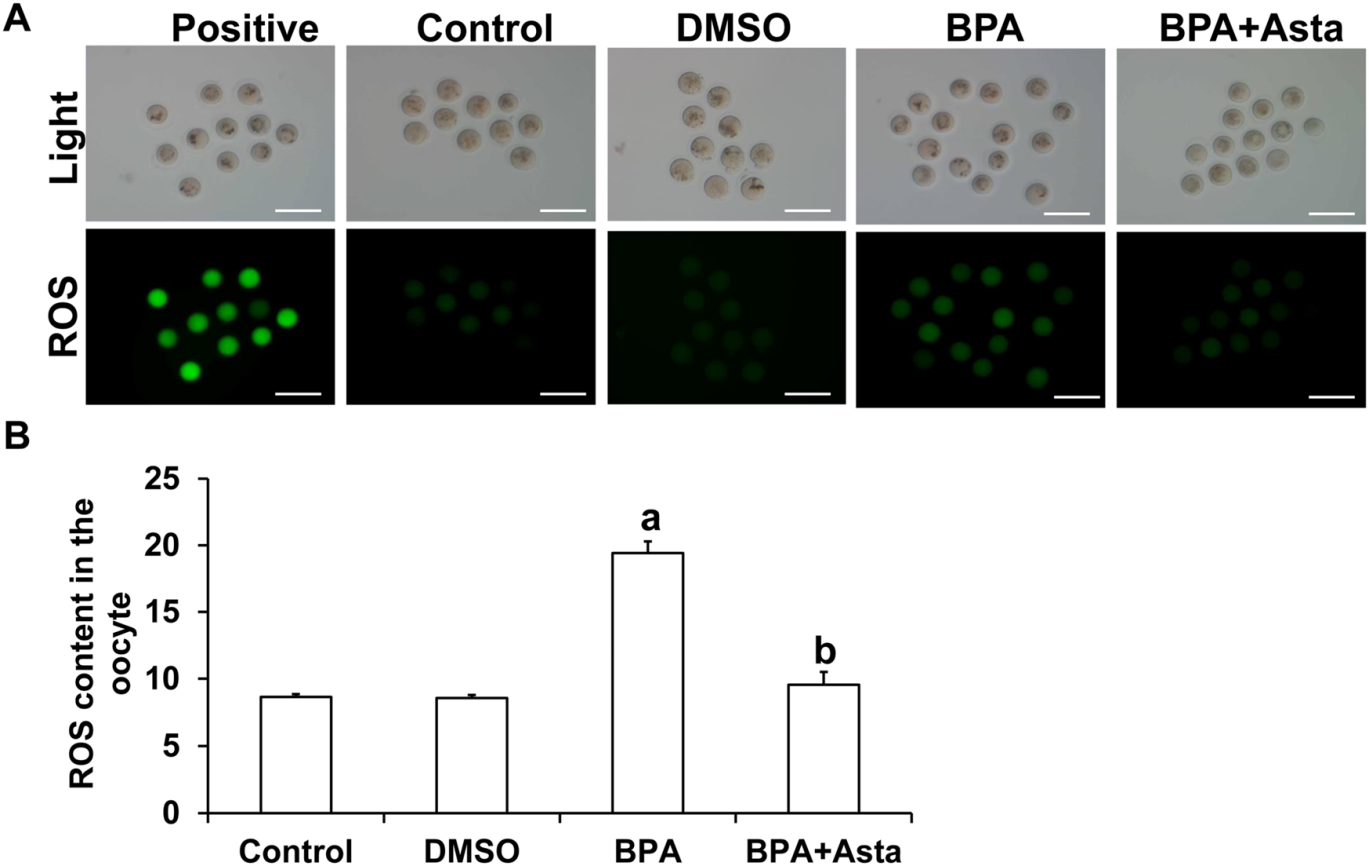
Analysis of ROS levels in oocytes. (**A**) Carboxy-H2DCF diacetate was used to detect the level of ROS in oocytes (Scale bar, 200 μm). (**B**) The average fluorescence intensity of ROS in oocytes was analyzed (N = 10). Compared with control, ^a^*P* < 0.05; and compared with BPA group, ^b^*P* < 0.05.

ROS staining was then performed on the oocytes discharged from D11. The stronger the green fluorescence in the oocytes, the higher the content of ROS (Fig. 6A). Statistically, the average fluorescence intensity of ROS in oocytes in the BPA group was significantly higher than the control group (*P* < 0.05) (Fig. 6B). The average fluorescence intensity of ROS in oocytes of the BPA+Asta group was significantly lower than the BPA group (*P* < 0.05) (Fig. 6B). These results suggest that astaxanthin reduces the production of ROS in oocytes.

### Astaxanthin increases mitochondrial membrane potential of oocytes and changes antioxidant and apoptosis-related gene expression levels

**Figure 7:**
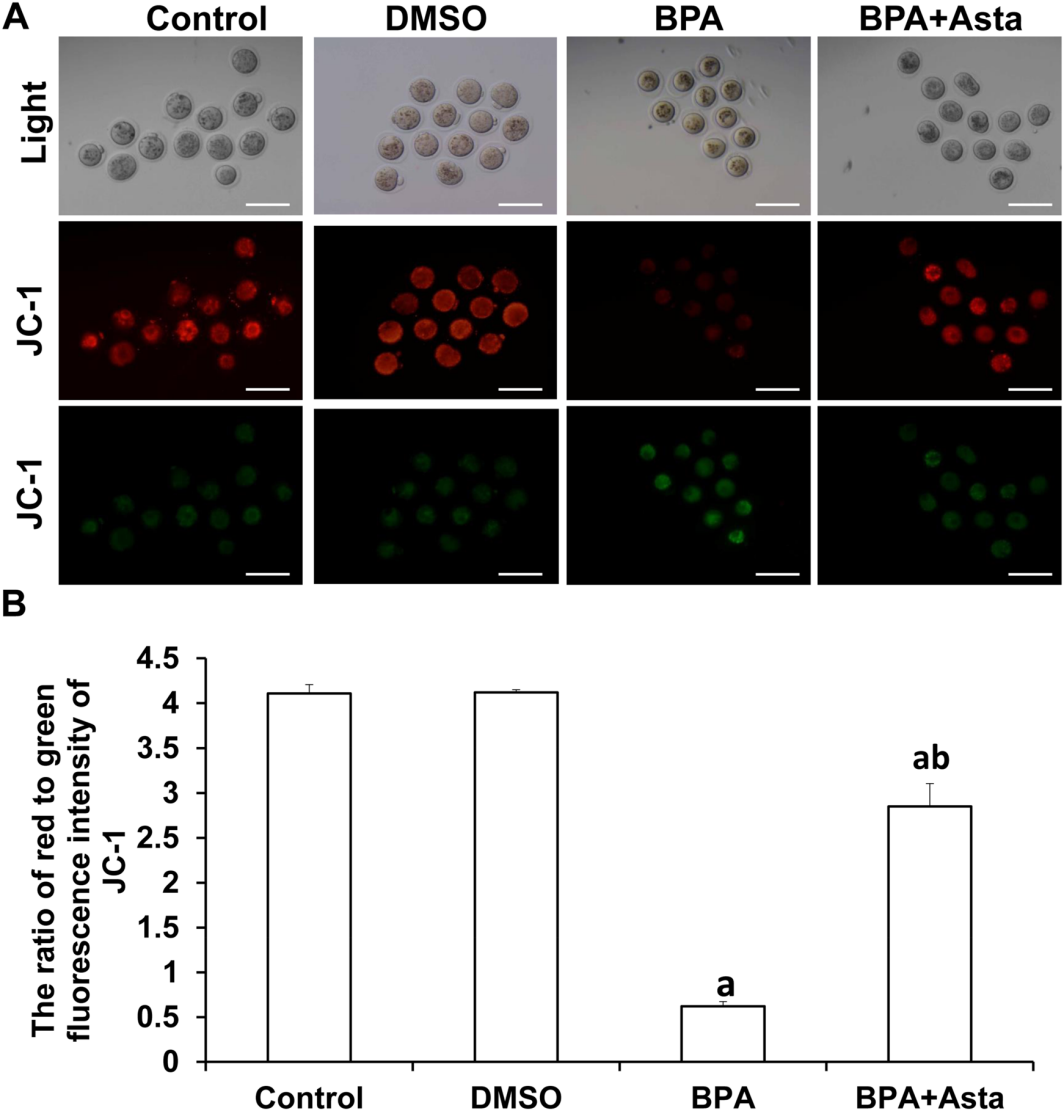
Analysis of mitochondrial membrane potential in oocytes. (**A**) JC-1 probe was used to detect the mitochondrial membrane potential in oocytes. JC-1 forms J-aggregates and produces red fluorescence under normal mitochondrial membrane potential (Scale bar, 200 μm). Under reduced or lost mitochondrial membrane potential, JC-1 exists in J-monomers and produces green fluorescence. (**B**) The average fluorescence intensity was analyzed in oocytes (N = 10). Compared with control, ^a^*P* < 0.05; and compared with BPA group, ^b^*P* < 0.05.

JC-1 probe was used to detect the mitochondrial membrane potential of oocytes. The higher ratio of red fluorescence to green fluorescence indicated that the mitochondrial membrane potential was normal, and the cells were in a healthy state (Fig. 7A). The ratio of red to green fluorescence of oocytes in the BPA group was significantly lower than the control group (*P* < 0.05) (Fig. 7B). The ratios of red to green fluorescence of oocytes in the BPA+Asta group were significantly higher than the BPA group (*P* < 0.05), and there was no significant difference between BPA+Asta group and control group. These results suggest that astaxanthin increases the mitochondrial membrane potential.

**Figure 8:**
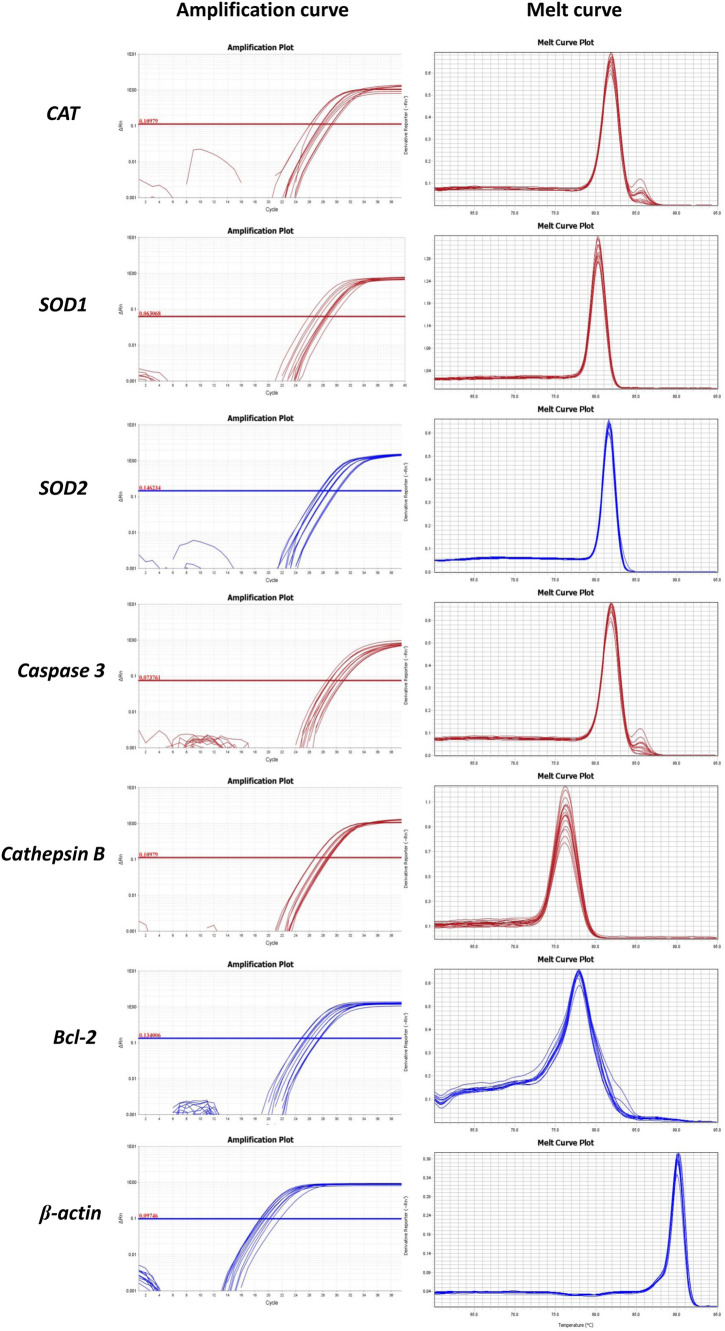
The amplification curves and melt curves in quantitative real-time PCR.

**Figure 9:**
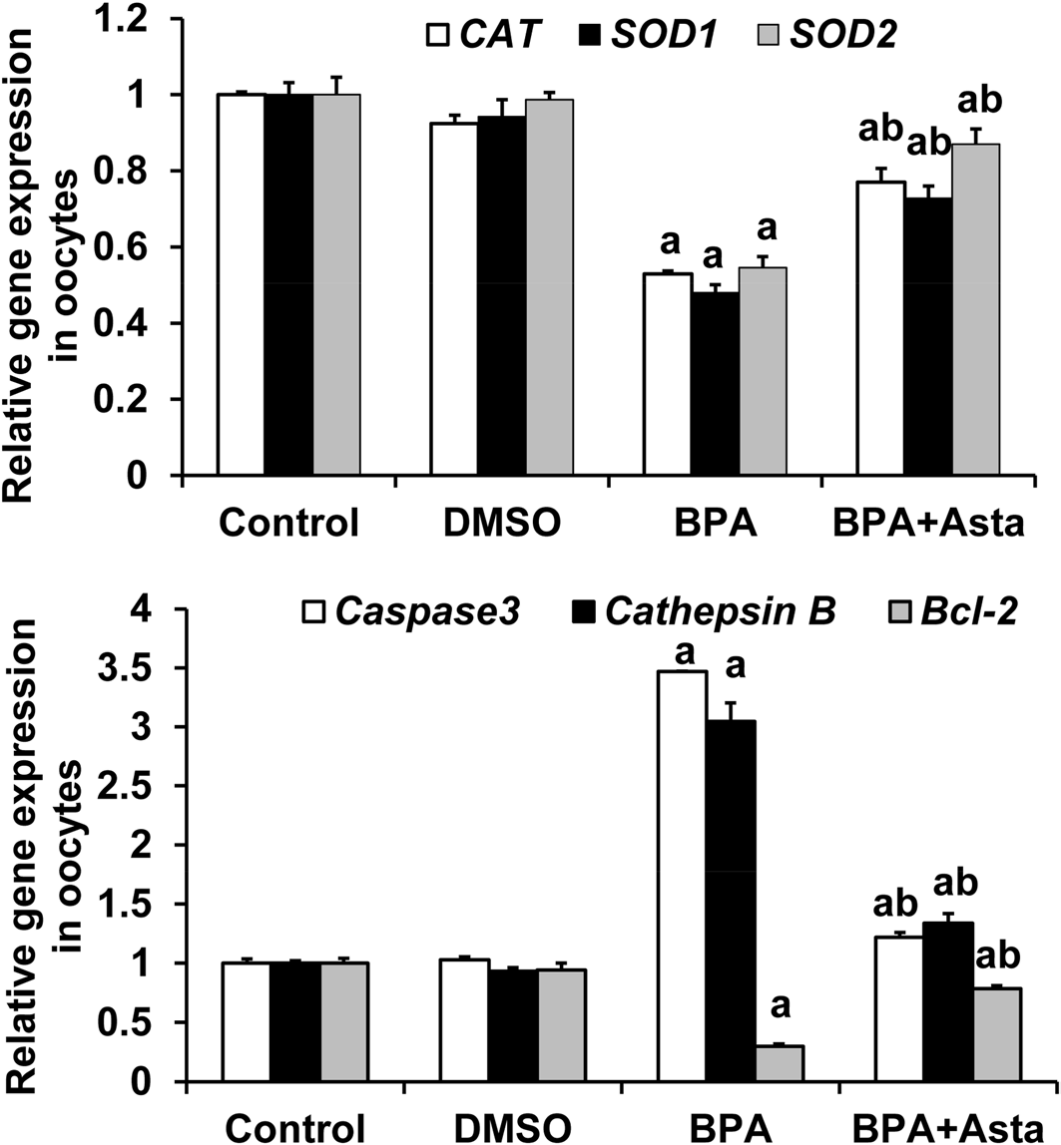
The mRNA expression levels of antioxidant genes (*CAT, SOD1,* and *SOD2*), apoptosis-related genes (*caspase 3* and *Bcl-*2) and *cathepsin B* in oocytes were detected with the quantitative real-time PCR. Compared with control, ^a^*P* < 0.05; and compared with BPA group, ^b^*P* < 0.05.

Quantitative real-time PCR was used to detect the expression of antioxidant genes (*CAT* (catalase), *SOD1* (superoxide dismutase 1), and *SOD2* (superoxide dismutase 2)), apoptosis-related genes (*caspase 3* and *Bcl-2*), and *cathepsin B* in oocytes (Fig. 8). Our results showed that the expression levels of antioxidant genes (*CAT, SOD1,* and *SOD2*) and the anti-apoptotic gene *Bcl-2* in the oocytes of the BPA group were significantly lower than the control group (*P* < 0.05) (Fig. 9). On the other hand, the expression levels of *caspase 3* and *cathepsin B* were significantly higher in BPA group than the control group (*P* < 0.05). Importantly, in BPA+Asta group, the expression levels of anti-oxidant genes (*CAT, SOD1,* and *SOD2*) and anti-apoptotic gene *Bcl-2* were significantly higher than those in the BPA group, while the expression levels of *caspase 3* and *cathepsin B* were significantly lower (*P* < 0.05). These results suggest that astaxanthin promotes the expression levels of antioxidant genes in oocytes and may inhibit oocyte apoptosis via regulating apoptosis-related genes.

## Discussion

More and more studies have shown that BPA is an important environmental substance that affects human reproduction^33–36^. It can inhibit the proliferation of ovarian granulosa cells, reduce the expression of connexins, and inhibit the expansion of CoCs and affect the meiosis recovery and maturation of ovarian oocytes^33–36^. However, these studies were performed in granulosa cells, oocytes or CoCs in the ovary separately^34–36^, which cannot better simulate the exposure to BPA in vivo. In this study, preantral follicles were separated. They were cultured in vitro for 11 days and exposed to BPA during the entire process of follicular development. The in vitro culture system of preantral follicles better maintained the connections between oocytes, granulose cells and theca cells. The effects of BPA on the development of follicles, the proliferation of granulosa cells and the maturation of oocytes were investigated simultaneously.

Oxidative stress has been considered to be an important harmful factor affecting the development of follicles, oocytes and embryos^37–39^. As a metabolic and endocrine disruptor, BPA can disrupt the oxidative balance in cells through direct or indirect ways, such as increasing mediators of oxidation reaction, reducing the production of antioxidant enzymes, damaging the mitochondrial function, and inducing apoptosis^40,41^. It can reduce the expression levels of antioxidant enzymes SOD, CAT, glutathione reductase and glutathione peroxidase in rat liver and epididymal sperm, and increase the contents of hydrogen peroxide in cells and the occurrence of lipid peroxidation^42,43^. In addition, cathepsin B is a lysosomal cysteine protease of the papain family, which can induce cell apoptosis, extracellular matrix degradation and intracellular protein catabolism^44^. The activity of cathepsin B is negatively correlated with the quality of oocytes and embryos^44^. When cathepsin B activity is reduced, the embryonic cell apoptosis is decreased and the developmental capacity and quality of embryos is improved^45^. Consistently, our results showed that 25 μM BPA significantly increased the levels of MDA in follicular culture medium, decreased the mitochondrial membrane potential of oocytes, the expression of antioxidant genes (*CAT, SOD1,* and *SOD2*) and the anti-apoptotic gene *Bcl-2*, and increased the *caspase 3* and *cathepsin B* expression levels. These findings suggest that BPA could induce the lipid peroxidation in the follicles, weaken the anti-oxidation ability of the oocytes, induce apoptosis, and reduce the quality of the oocytes.

Astaxanthin is a strong fat-soluble antioxidant that can reduce lipid peroxidation and DNA damage in oocytes and embryos^28^. It is found that astaxanthin significantly increased the expression levels of antioxidant gene *GPX4* in aging pig oocytes, the levels of reduced glutathione in oocytes, and the mRNA levels of *BCL2L1* and *SURVIVIN*, but significantly reduced the cathepsin B activity and *caspase-3* mRNA levels, thereby improving the developmental ability of aging oocytes and parthenogenetic embryos^30^. For frozen pig oocytes, astaxanthin could significantly increase the levels of glutathione and mitochondrial activity in the cells, thus improving the anti-oxidation and development of the frozen oocytes^46^. In this study, astaxanthin was used to alleviate the oxidative damages of BPA to follicular development. Our results showed that the mitochondrial membrane potential, the expression levels of antioxidant genes (*CAT, SOD1*, *and SOD2*) and anti-apoptotic gene *Bcl-2* in oocytes of the BPA+Asta group were significantly increased. The levels of MDA in the culture medium and the expression levels of *caspase 3* and *cathepsin B* in oocytes were significantly reduced. Thus, we suppose that astaxanthin could improve the antioxidant capacity of oocytes, inhibit oocyte apoptosis, and alleviate the damages of BPA to the development of oocytes. In addition, astaxanthin significantly increased the survival rate, antrum formation rate, and oocyte maturation rate of follicles, and increased the expression levels of PCNA in granulosa cells in the late stage of follicular development, promoting the proliferation of granulosa cells.

Oxidative stress is mainly caused by excessive ROS production or defects in the antioxidant mechanism in the cells^47^. ROS would react with intracellular macromolecular substance, such as lipids, proteins and DNA, causing intracellular enzyme inactivation, destruction of cell membrane integrity, abnormal mitochondrial function, and DNA fragmentation^47^. ROS can also block and delay the development of preimplantation embryos, and reduce the in vitro development rate of preantral follicles^31^. BPA can significantly increase the lipid peroxidation and ROS production of ovarian tissue, and affect the normal development of follicles and oocytes^5^. BPA can also significantly increase the ROS levels in pig embryos, causing intracellular cytochrome C release, mitochondrial and DNA damages, and apoptosis^48^. It is shown that antioxidants can reduce the production of ROS^28^, increase the expression levels of antioxidant genes in cells, and improve the maturation, fertilization and early embryonic development capabilities of oocytes^49^. Jia et al*.*^30^ reported that 2.5 μM astaxanthin significantly reduced the production of ROS in oocytes and improved the quality of oocytes^30^. In this study, we have obtained similar results, which showed that the BPA treatment significantly increased the ROS levels in oocytes and reduced the maturation rate of oocytes. On the other hand, astaxanthin significantly reduced the production of ROS in oocytes, significantly increased the maturation rate of oocytes, and significantly relieved the oxidative stress damage in oocytes caused by BPA.

The whole process of follicular development is accompanied by the continuous proliferation of granulosa cells, and the steroid hormones synthesized and secreted by granulosa cells and theca cells further stimulate the development of follicles^50^. BPA is reported to inhibit the proliferation of granulosa cells, interfere with the synthesis of steroid hormones, and interfere with the follicular development^50^. Since the volume of oocytes hardly changes during the development of preantral follicles with the diameter of 110–130 μm^50^, the adherence area of follicles may reflect the proliferation of granulosa cells. Additionally, PCNA is involved in regulating DNA synthesis and is an important indicator of cell proliferation^50^. In this study, BPA was added to the mouse preantral follicle culture medium, and our results showed that the adherence area of the follicles in the BPA group was significantly reduced from D4, and the PCNA expression levels of D8 and D10 granulosa cells were also significantly lower than the control group. This suggests that BPA may inhibit the proliferation of granulosa cells. Zhou et al*.*^51^ found that in the in vitro culture of granulosa cells, BPA inhibited the proliferation of rat follicular theca cells and granulosa cells, and reduced the secretion of estrogen and progesterone. Abdel-Ghani et al*.*^31^ showed that astaxanthin (500 μM) significantly increased the synthesis and secretion of estradiol in follicles, and decreased the synthesis and secretion of progesterone. Kamada et al*.*^52^ have found that low concentration (0.1–10 nM) of astaxanthin could increase the synthesis of progesterone in the luteal cells, while high concentration (1000 nM) of astaxanthin could inhibit the synthesis of progesterone. Therefore, we speculate that the effects of astaxanthin on progesterone synthesis may not be caused by the antioxidant effect. In this study, 2.5 nM astaxanthin was added into the in vitro culture medium of preantral follicles, which significantly increased the secretion of estrogen and progesterone in the follicles, and increased the expression levels of ERα in the granulosa cells, and the estrogen secreted by the follicles. The secreted estrogen would bind to the ER of the follicle to further stimulate the production of estrogen^53^. Astaxanthin significantly alleviated the interference of BPA on the synthesis of follicular steroid hormones. However, whether it works through the mechanism of alleviating antioxidant effects is still unclear, and further studies are warranted.

In conclusion, our results showed that astaxanthin improved the antioxidant capacity of oocytes by increasing the expression levels of antioxidant genes in oocytes, reduced the lipid peroxidation of follicles and the production of ROS, and protected oocytes against BPA-induced damages of the cell mitochondrial membrane potential, therefore promoting the proliferation of granulosa cells, improving the development rate of follicles and the maturation rate of oocytes, and significantly alleviating the oxidative stress damages to the follicles. Astaxanthin also increased the secretion of estrogen and progesterone in follicles, and relieved the inhibitory effects of BPA on the synthesis of steroid hormones. However, it was still necessary to further clarify the relationship between hormone synthesis and the antioxidant activity of astaxanthin. Therefore, as a high-efficiency antioxidant, astaxanthin is expected to become an antioxidant drug for the female reproductive system, protecting germ cells from oxidative stress damage and preventing premature ovarian failure and other diseases.

## Methods

### Study animals

The 14-day female Kunming mice (n = 90) were purchased from the Yisi Experimental Animal Technology Co., Ltd. (Changchun, Jilin, China). The mice were housed in a temperature-controlled room (22 ± 2 °C), with a light cycle of 12 h light/12 h dark, and free access to drinking and eating. All the animal experimental procedures were approved by the ethics committee of the Jilin Medical College. All experiments were performed in accordance with relevant guidelines and regulations. The study was carried out in compliance with the ARRIVE guidelines.

### Preparation of BPA and astaxanthin

DMSO (D2650; Sigma, St. Louis, MO, USA) was used to dissolve BPA (239658; Sigma) and astaxanthin (SML0982; Sigma). BPA and astaxanthin stock solutions at 25 mmol/L and 2.5 μmol/L were prepared. Before use, 1 µL BPA or astaxanthin stock solution was added into 1 mL in vitro culture medium for follicle to make the working concentration of 25 µmol/L or 2.5 nmol/L. Previous studies have found that 25 µmol/L BPA has germ cell toxicity^44^, while 2.5 nmol/L astaxanthin has significant antioxidant activity^54^. In the solvent group, 2 μL DMSO was added into 1 mL in vitro culture medium for follicle to make the final concentration of 0.2%.

### The in vitro culture and treatment of mouse preantral follicles

The in vitro culture of mouse preantral follicles was performed according to a previous method published by Liu et al.^55^ Briefly, the 14-day Kunming female mice were sacrificed by cervical dislocation. Their bilateral ovaries were quickly removed to L-15 working solution (containing 10% FBS) (11415114; Gibco, GrandIsland, NY, USA). A 26G needle was used to mechanically isolate the follicles. The preantral follicles were selected according to the Pedersen and Peter's grading criteria for grade 4 or 5a follicles with a diameter of 110–130 μm^56^. Namely, the preantral follicles consisted of a round zona pellucida-wrapped oocyte in the middle of the follicle, 2–4 layers of granulosa cells, intact basement membrane and several theca cells attached to the basement membrane. These follicles are growing follicles. The selected preantral follicle was placed in a droplet of α-MEM culture medium (12571063; Gibco), which contained 5% FBS (10091155; Gibco), 1% ITS (41400045; Gibco) and 0.1 IU/ml r-FSH; Gonal-F, Serono), and cultured in a 37 °C, 5% CO_2_ incubator, for 10 days.

In the BPA group, 25 µmol/L BPA was added into the culture medium, and in the DMSO group, 0.2% DMSO was added. In the BPA+Asta group, 25 µmol/L BPA and 2.5 nmol/L astaxanthin was added into the culture medium. The medium was semi-quantitatively replaced every other day. The supernatant was collected and stored at − 20 °C for further analysis. The control group was untreated. The growth of follicles on day 0 (D0), day 2 (D2), day 4 (D4), day 6 (D6), day 8 (D8) and day 10 (D10) was observed and recorded, with the cellSens microscopic image software of the Olympus IX-83 microscope. On D2, the follicles that began to grow adherently were defined as survival follicles. The survival rate of the cultured follicles was calculated as the ratio of survival follicles to total cultured follicles. On D10, the antrum was formed in the follicle. The antrum formation rate was defined as the ratio of the follicles with antrum to the survival follicles. On D10, the culture medium containing 2.5 U/ml hCG (Livzon Pharmaceutical Factory) was added. After 14–16 h, mature follicles ruptured and ovulated naturally. The ovulation rate was defined as the ratio of the follicles that ovulated to the follicles with antrum. The ovulated CoCs were denuded. MII oocytes with the first polar body were mature oocytes. Maturation rate was defined as the ratio of the mature oocytes to the total denuded oocytes. The Image J software was used to analyze the adherence area of follicles on the petri dish, and 10 follicles were analyzed in each group.

### ELISA

The levels of estrogen and progesterone in D10 follicle culture medium were detected with ELISA kits (BPE20376 and BPE20381; Shanghai Lengton Bioscience Co., Ltd., Shanghai, China), according to the kit instructions. The absorbance value was measured at 450 nm using a SpectraMax Absorbance Reader (Molecular Devices).

### Western blotting analysis

In each group, granulosa cells were obtained from 40 follicles on Day 8 or 10 of the in vitro culture. Protein was extracted from granulosa cells with RIPA strong lysate (containing 1% PMSF) (P0013B and P1006; Beyotime) and separated by 12% SDS-PAGE. Protein was transferred onto the PVDF membrane, which was then blocked with 5% skimmed milk for 1 h. Then the membrane was treated with primary antibodies against proliferating cell nuclear antigen (PCNA, ab92552: Abcam, Cambridge, MA, USA), estrogen receptor α (ERα) (ab32063; Abcam) or β-actin (ab8226; Abcam) at 4 °C overnight. After washing with PBST, the membrane was incubated with HRP-conjugated goat anti-rabbit secondary antibody (31210; Thermo Scientific Pierce) at room temperature for 2 h. Color development was performed with the enhanced chemiluminescence. The protein bands were scanned using the ChemiDOC XRS + imaging systems (Bio-Rad Laboratories, Hercules, CA, USA). β-actin was used as an internal control for protein loading. Image J image analysis software was used to analyze the relative expression levels of PCNA and ER-α based on the density of β-actin. The original images of full-length blots are in the Supplementary Information.

### MDA content determination

MDA has been widely used as an indicator of lipid peroxidation^32^. It can react with thiobarbituric acid (TBA) at higher temperature and acidic environment to form a red MDA-TBA adduct, which could be detected by colorimetry^57^. The Lipid Peroxidation MDA Assay Kit (S0131S; Beyotime) was used to determine the contents of MDA in D10 follicle culture medium. The specific method was as follows^57^: D10 follicle culture medium frozen at − 20 °C was thawed, and centrifuged at 1600 × g at 4° C for 10 min. The supernatant was collected. Totally 200 µl TBA reagent was added to the supernatant of 100 µl follicle culture medium. The mixture was heated in boiling water bath for 15 min. After cooling, the reaction solution was centrifuged at 1000 × g for 10 min, and the supernatant was collected. Totally 200 μl supernatant was added to a 96-well plate. The absorbance value at 532 nm was measured using the SpectraMax Absorbance Reader (Molecular Devices). The above experiment was repeated five times.

### ROS level detection with H2DCF diacetate

Carboxy-H2DCF diacetate was used to assess ROS levels in oocytes as previously reported^54,58^. After entering cells, it is hydrolyzed by intracellular esterases to generate DCFH. DCFH is non-fluorescent and impermeable to cell membranes. Intracellular ROS can oxidize non-fluorescent DCFH to generate fluorescent DCF. The level of intracellular ROS could be determined by detecting the fluorescence of DCF. After culture with HCG for 14–16 h, the CoCs were obtained. After removing the cumulus cells, the obtained oocytes (day 11, D11) were incubated in the preheated M2 medium containing 10 mM carboxy-H2DCF diacetate (Cat #S0033; Beyotime) at 37 °C for 30 min in dark. After washing with M2 medium for 3 times under low light, the oocytes were placed in the M2 medium in the 35-mm dish and were observed with the cellSens microscopic imaging software of the Olympus IX-83 microscope. The oocytes of the positive group were incubated in the M2 medium containing Rosup (1:1000 dilution; Cat #S0033; Beyotime) for 20 min at 37 °C, and then in 10 mM carboxy-H2DCF diacetate (Cat #S0033; Beyotime) at 37 °C for 30 min. In each experiment, the green fluorescence signal was obtained by the cellSens microscopic imaging software of the Olympus IX-83 microscope with the same settings. Image J software (NIH image, Bethesda, MD) was used to quantify the fluorescence intensity. Briefly, the captured images were converted to 8-bit images, and then a threshold was set for the green channel of fluorescence. The fluorescence intensity of each oocyte was calculated. Totally 10 oocytes were analyzed from each group.

### Mitochondrial membrane potential detection with JC-1

The JC-1 probe was used to detect the mitochondrial membrane potential according to previously published methods^59,60^. Briefly, after washing with MII medium for 3 times, the oocytes were placed in a culture medium, containing 0.5 µmol/L JC-1 (Invitrogen, Grand Island, NY, USA) in a 37 °C, 5% CO_2_ incubator for 30 min. JC-1 forms J-aggregates and produces red fluorescence under normal mitochondrial membrane potential. When there is cell apoptosis, the mitochondrial membrane potential would be decreased or even lost, and JC-1 exists in J-monomers and produces green fluorescence. Fluorescence was captured with the the cellSens microscopic image software using the Olympus IX-83 fluorescence microscope. The ratio of red and green fluorescence intensities of oocytes reflected the mitochondrial membrane potential. Totally 10 oocytes were analyzed from each group.

### Quantitative real-time PCR

Totally, 60 oocytes were analyzed by quantitative real-time PCR. Total RNA was extracted from oocytes using the Rneasy Micro Kit (Qiagen, Hilden, Germany). Reverse transcription was performed to obtain cDNA in a 20 μL reverse transcription system, consisting of 1 μL random primers, 1 μL Oligo dT Primer, 4 μL Reverse Transcription buffer, and 1 IU/mL PrimeScriptTEMRT Enzyme Mix I (TaKaRa, Dalian, China). Quantitative real-time PCR was performed on the iQ5 Multicolor Real-time PCR Detection System (Bio-RAD), using the SYBR Premix Ex Taq (Takara, Dalian, Liaoning, China). Primer sequences were listed in Table 2. PCR reaction system consisted of Premix Ex TaqTMII, forward/reverse primers and cDNA template. PCR reaction conditions were as follows: 95 °C for 30 s; 95 °C for 5 s, 57–60 °C (depending on the primers used) for 20 s, 72 °C for 30 s, for totally 40 cycles. Three replicates were performed for each reaction. The 2^-△△Ct^ method was used to calculate the relative expression levels of target genes. β-actin was used as internal reference.

**Table 2:** Primer sequences for quantitative real-time PCR.

Gene	Primer sequences (5′to 3′)		Annealing temperature
	Forward	Reverse	
*CAT*	TTACCCCAACAGCTTCAGCGCA	GGCAATGTTCTCACACAGGCGT	58
*SOD1*	AGCATGGGTTCCACGTCCATCA	ACCGTCCTTTCCAGCAGTCACA	58
*SOD2*	ATAATGTTGTGTCGGGCGGCGT	TCGGTGGCGTTGAGATTGTTCACG	60
*Caspase 3*	TGGCATTGAGACAGACAGTGGGA	TGCGCGTACAGCTTCAGCAT	57
*Cathepsin B*	TGGCAAGATTTGGACGACTGGACC	ACAGCAGGCACTACAAACCGCA	60
*Bcl-2*	ACCGTCGTGACTTCGCAGAGAT	TGTGCAGATGCCGGTTCAGGTA	58
*β-actin*	TGTTACCAACTGGGACGACA	CTGGGTCA TCTTTTCACGGT	58

CAT, catalase; SOD1, superoxide dismutase 1; SOD2, superoxide dismutase 2; and Bcl-2, B-cell lymphoma-2.

### Statistical analysis

Data were expressed as mean ± SD. The SPSS 17.0 statistical software was used for statistical analysis. Data were analyzed with the one-way ANOVA and the LSD post doc test. *P* < 0.05 was considered to be statistically significant.

### Ethics approval

All the animal experimental procedures were approved by the ethics committee of the Jilin Medical College. All experiments were performed in accordance with relevant guidelines and regulations. The study was carried out in compliance with the ARRIVE guidelines.

## Supplementary Information

Supplementary Information.

## Data Availability

The datasets generated during and/or analysed during the current study are available from the corresponding author on reasonable request.
