# Supplementary figures and images for: Astaxanthin improves the development of the follicles and oocytes through alleviating oxidative stress induced by BPA in cultured follicles
Source: Sci Rep. 2022 May 12;12:7853. doi: 10.1038/s41598-022-11566-1 (PMC9098901; doi:10.1038/s41598-022-11566-1)

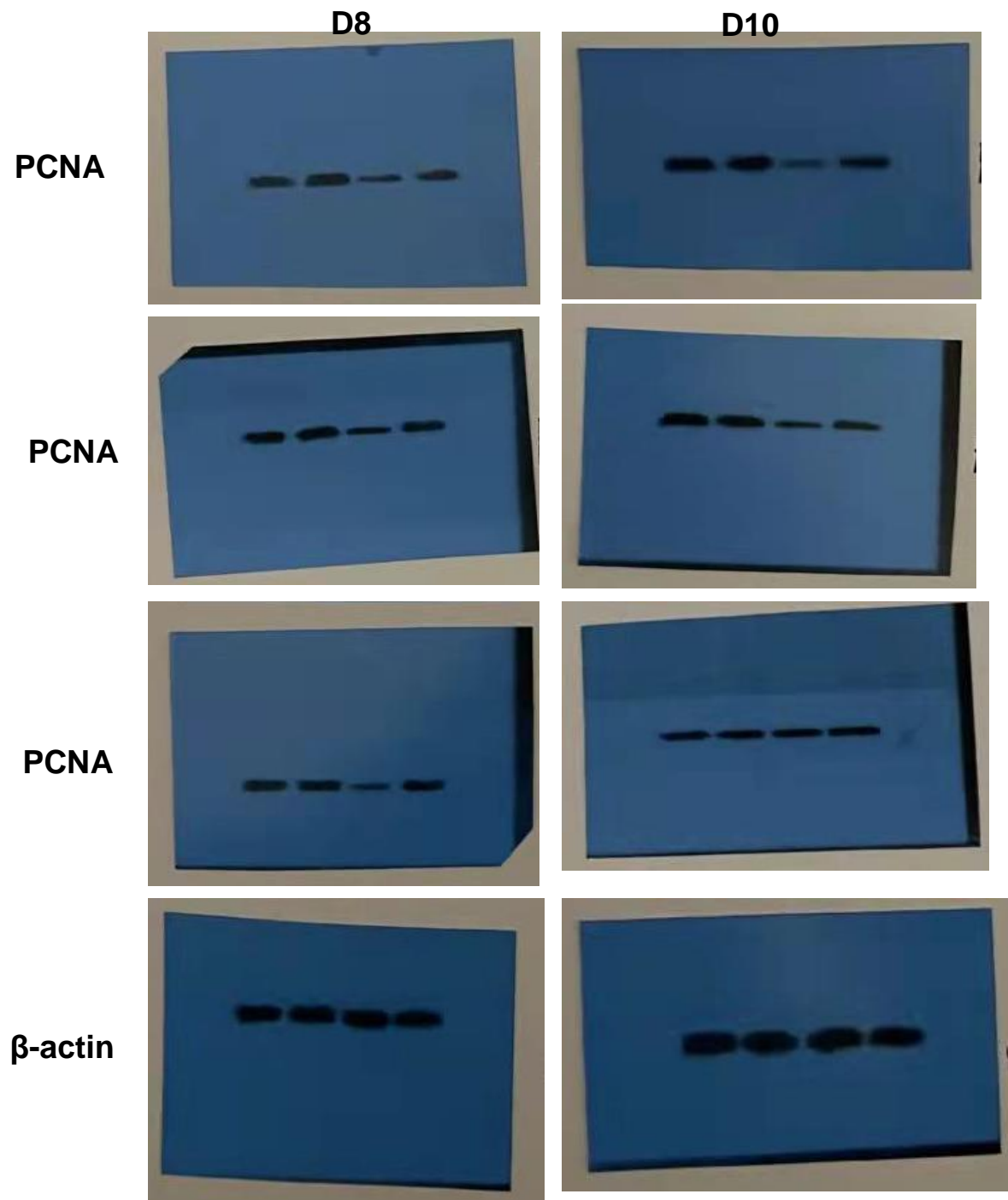

**D10**

**ER $\alpha$**

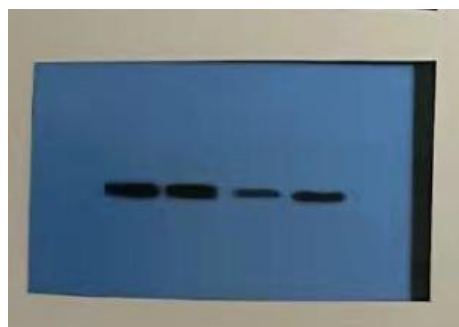

**ER $\alpha$**

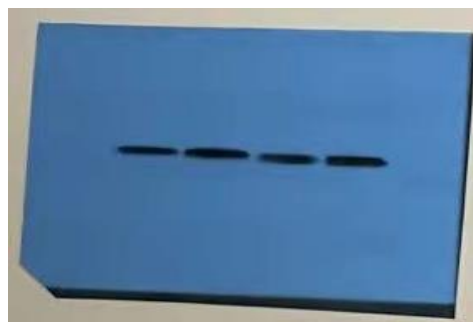

**ER $\alpha$**

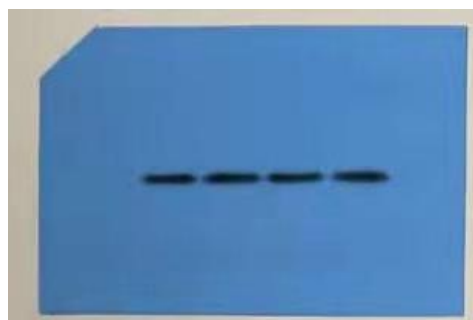

**$\beta$ -actin**

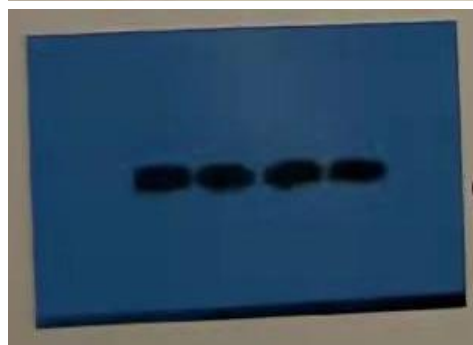

Supplement: Supplementary file 1 — Supplementary Information. [file 41598_2022_11566_MOESM1_ESM.pdf]
